# Role of national regime ideology for predicting biodiversity outcomes

**DOI:** 10.1111/cobi.14314

**Published:** 2024-08-06

**Authors:** Jacob Jones, Andrea S. Griffin, Frank W. Agbola, Matt W. Hayward

**Affiliations:** ^1^ Conservation Science Research Group, School of Environmental and Life Sciences University of Newcastle Callaghan New South Wales Australia; ^2^ Newcastle Business School University of Newcastle Callaghan New South Wales Australia; ^3^ Asia Pacific Research Centre, College of Human and Social Futures University of Newcastle Callaghan New South Wales Australia; ^4^ Centre for African Conservation Ecology Nelson Mandela University Gqeberha South Africa

**Keywords:** conservation communications, conservatism, democracy, government, nationalism, protected areas, socialism, threatened species, áreas protegidas, comunicación de la conservación, conservadurismo, democracia, especie amenazada, gobierno, nacionalismo, socialismo

## Abstract

The rapid decline of global biodiversity has engendered renewed debate about the social, economic, and political factors contributing to it. Specifically, there is little understanding of the role that political ideology within a country (e.g., nationalism, conservatism, socialism) plays in determining biodiversity outcomes. We used negative binomial generalized linear models to investigate the importance of national regime ideology in predicting threatened animal species and protected area establishment compared with other factors that affect biodiversity outcomes, such as gross domestic product, inequality, and democracy. For threatened animals, the model with the highest Akaike weight suggested adverse biodiversity outcomes arose from larger gross domestic product (β = 0.120, *p* < 0.001). However, nationalism (β = 0.371, *p* < 0.01) and socialism (β = 0.293, *p* < 0.05) were also significantly associated with increased proportions of threatened species. For protected areas, the model with the highest Akaike weight suggested increases in democracy (β = 0.880, *p* < 0.001) led to a rise in relative protected area estate. Conservative regime ideology was also associated with greater protected area estate, although this did not increase the weight of evidence in support of the best models. These findings highlight the relevance of political ideology for predicting biodiversity outcomes at a national scale and illustrate opportunities to tailor policies and advocacy to promote biodiversity conservation more effectively. By targeting appropriate messaging and political advocacy, conservationists can improve the likelihood that politicians and their nations will participate in positive biodiversity actions.

## Abstract (es)

El papel de la ideología del régimen nacional para la predicción de resultados de biodiversidad

**Resumen:**

El rápido declive de la biodiversidad mundial ha suscitado un renovado debate sobre los factores sociales, económicos y políticos que contribuyen a él. En concreto, se conoce poco el papel que desempeña la ideología política dentro de un país (por ejemplo, el nacionalismo, el conservadurismo o el socialismo) a la hora de determinar los resultados en materia de biodiversidad. Utilizamos modelos lineales generalizados binomiales negativos para investigar la importancia de la ideología del régimen nacional a la hora de predecir las especies animales amenazadas y el establecimiento de áreas protegidas en comparación con otros factores que afectan a los resultados de la biodiversidad, como el producto interno bruto, la desigualdad y la democracia. En el caso de los animales amenazados, el modelo con la mayor ponderación de Akaike sugirió que los resultados adversos para la biodiversidad se debían a un mayor producto interno bruto (β = 0,120, p < 0,001). Sin embargo, el nacionalismo (β = 0,371, p < 0,01) y el socialismo (β = 0,293, p < 0,05) también se asociaron significativamente con una mayor proporción de especies amenazadas. En el caso de las áreas protegidas, el modelo con la mayor ponderación de Akaike sugirió que el aumento de la democracia (β = 0,880, p < 0,001) conducía a un aumento de la extensión relativa de las áreas protegidas. La ideología conservadora del régimen también se asoció con una mayor superficie de áreas protegidas, aunque no aumentó el peso de la evidencia en apoyo de los mejores modelos. Estos resultados resaltan la importancia de la ideología política para predecir los resultados de la biodiversidad a escala nacional e ilustran las oportunidades de adaptar las políticas y la defensa para promover la conservación de la biodiversidad de manera más eficaz. Si se orientan los mensajes y la promoción política de forma adecuada, los conservacionistas pueden mejorar la probabilidad de que los políticos y sus naciones participen en acciones positivas para la biodiversidad.

## INTRODUCTION

The global decline of biodiversity is a pressing challenge that will have long‐lasting consequences for humans and ecosystems (Rockström et al., 2009). Factors such as deforestation (Betts et al., 2017), invasive species introductions (Doherty et al., 2016), and climate change (Titley et al., 2021) are frequently recognized as significant physical drivers of biodiversity decline. However, for effective and sustainable conservation practice, it is equally important to consider the underlying social, economic, and political factors that encourage these drivers (Hoffmann, 2004; Rydén et al., 2020). By gaining a deeper understanding of these underlying factors, global conservation interventions can be designed more effectively, and the focus can shift toward prevention rather than cure.

There is considerable uncertainty regarding the social, economic, and political correlates of biodiversity loss. For example, many cross‐national studies have explored factors such as living standards, economy size, population, or the presence of environmental nongovernment organizations (ENGOs) with mixed results (Hoffmann, 2004; McKinney et al., 2009, 2010; Shandra et al., 2009). Economic inequality and poor governance have emerged as consistent predictors of biodiversity loss, although there is still debate about the underlying causal mechanisms (Barrett et al., 2006; Kubiszewski et al., 2023). A recent review investigating the relationship between democracy and biodiversity has also found mixed results (Rydén et al., 2020). This suggests that work remains to be done to understand the collection of social, economic, and political factors affecting biodiversity outcomes. Identifying and understanding these factors is critical for developing and implementing more effective national and global interventions to address biodiversity loss.

Political ideology is another influential factor for environmental agenda‐setting and decision‐making (Jahn, 1998; Wen et al., 2016), yet it has not been empirically linked to biodiversity outcomes at cross‐national scales. At the individual level, political ideology has provided strong evidence as an important predictor of environmental and biodiversity policy support, although the relationship is mediated by other economic or cultural factors (Cruz, 2017; Czech & Borkhataria, 2001; Nawrotzki, 2012). At governmental levels, regime ideology has also predicted mixed results for broader environmental policies and outcomes. For example, left wing governments are more likely to promote environmental quality over economic performance (Wen et al., 2016), although whether left wing governments deliver better environmental outcomes is still subject to debate (Tawiah, 2022). Nevertheless, the influence of regime ideology has not been quantified specifically for biodiversity outcomes, either in terms of physical risk to national biodiversity or in terms of governmental commitment to conservation.

We explored this knowledge gap for the first time by quantifying whether national regime ideology is an important factor to consider in predicting the risk to biodiversity and the commitment to conserving it among 165 nation‐states. We also extended previous research by adopting a multidimensional approach to regime ideology. Although the left–right spectrum offers a quick and straightforward way of categorizing the ideology of governments at subnational scales, due to various historical and cultural factors, the meaning of left–right ideology is not always consistent at global scales (Gindler, 2020; Nawrotzki, 2012). Instead, by focusing on ideologies, such as conservatism, socialism, and nationalism, that have more universal understanding than left and right, we aimed to draw more meaningful conclusions as to the relative influence of regime ideology on biodiversity. In the present study, *multidimensional ideology* means a national regime can be socialist and nationalist, nationalist and conservative, or any mixture of these (Tannenberg et al., 2019).

National regime ideology is “an officially codified set of beliefs used to justify a particular set of social, political and economic relations” (Tannenberg et al., 2019, p. 8). For national governments, the codified set of beliefs can be used to justify a particular set of political processes and institutions. Depending on the ideology embraced, governments may prioritize certain values over others. For instance, conservative regimes tend to prioritize private property rights and open markets, whereas socialist regimes tend to prioritize societal equality and wealth redistribution (Gindler, 2020). Through these institutional and political processes, regime ideology is a central component of the decision‐making apparatus that guides a national government's response to social, economic, and environmental challenges (Wen et al., 2016).

There are various theoretical links between regime ideology and biodiversity. These links are rooted in different worldviews that guide a national government's preferred management style for conservation and influence the appetite for trade‐offs between biodiversity and other socioeconomic factors. For example, conservative ideology, which is often evident in many Western democracies (Tannenberg et al., 2019), is strongly linked to capitalism and economic instruments as a framework for conserving nature (MacDonald, 2010). Within this framework, dominant conservation models include ecotourism, biodiversity offsetting, trophy hunting, and the broader neoliberal conservation paradigm (Büscher et al., 2012; Penca, 2013). At the core of this framework is conserving nature through commodification and valuation in economic terms (Apostolopoulou et al., 2021). In principle, this allows environmental protection and economic growth to coexist, which is important for biodiversity given conservative ideology's traditional assertion that environmental and economic outcomes are mutually exclusive (Harring & Sohlberg, 2016; Wen et al., 2016).

Often portrayed at the opposite end of the political spectrum is socialism. Socialism is less focused on the accumulation of capital and open markets. However, it has still faced criticism from ecologists for its emphasis on productivism and for an anthropocentric valuation of nature (Kluvánková‐Oravská et al., 2009; Kopnina et al., 2018; Löwy, 2005). For example, in Madagascar, socialist policies of the 1970s promoted the conversion of natural areas into productive land to raise standards of living of its citizenry (Horning, 2008), and in Central and Eastern Europe, protected areas existed, but socialist centralized management and enforcement were so poor that many protected areas became “*de facto* open access” (Bromley, 1992, p. 13; Chobotová, 2013). However, contemporary postcapitalist conservation movements are increasingly calling for the merging of social justice and biodiversity as dual motives required for sustainable ecological futures (Baldauf, 2020; Büscher & Fletcher, 2019). There is a focus on remedying land dispossession (particularly colonial‐era fortress conservation in the Global South) and neoliberal conservation legacies and moving toward alternate forms of ecological valuation and human–nature coexistence. Others have argued that these modern human‐orientated approaches to conservation align most closely with socialist ideological principals (Kopnina et al., 2018).

Other ideologies, such as nationalism, have been linked to conservation through national identity and territorial security, which can manifest through factors such as neocolonialism and increasing militarization (Haila, 2012; Hodgetts et al., 2019). For global biodiversity outcomes, these effects are often pronounced through geopolitical relations between the Global North and Global South because historically wealthy nations, such as the United Kingdom, France, and the United States, and more recently China, compete for economic and political influence and access to natural resources in socioeconomically poor but biodiversity‐rich regions (Büscher et al., 2012; Hodgetts et al., 2019). Despite countermovements of natural resource sovereignty and economic nationalism that saw the Global South renegotiate the terms of the global world order, the rules of globalization and neoliberalism popularized by the Global North have largely cemented geopolitical inequalities (Frame, 2022; Frey et al., 2019). Therefore, nationalist regime ideology may also reinforce attitudes toward socioeconomic individualism and reduce commitment to addressing environmental issues of international concern requiring transnational cooperation, particularly at a perceived cost to national interests (Hale, 2020; Hodgetts et al., 2019; Raustiala, 1997).

Regime ideology could therefore offer conservation advocates new perspectives through which to direct conservation resources more effectively or promote conservation interventions in the right way. To evaluate the merit of this idea, we aimed to determine whether national regime ideology helps explain national threat to biodiversity and commitment to conservation. We considered the threat to biodiversity as the number of threatened animal species in a country. Commitment to conservation was measured as the total protected area estate, as protected areas are critical tools for slowing biodiversity decline worldwide (Rodrigues & Rouyer, 2023). We investigated these 2 dimensions of biodiversity and conservation because we suspected their relationships with regime ideology were independent and developed a broad set of national‐level predictions. First, conservative ideology would be associated with higher rates of threatened animals and larger proportions of protected area estate due to conservatism's connections to resource‐intensive capitalism and neoliberal conservation paradigms such as biodiversity markets or game hunting (Apostolopoulou et al., 2021; Penca, 2013). Second, socialist ideology would be associated with lower rates of threatened animals and smaller proportions of protected area estate because of a greater emphasis on social–environmental justice and prioritizing participatory conservation over more traditional “fortress conservation” models (Baldauf, 2020; Kopnina et al., 2018). Third, nationalist ideology would be associated with higher rates of threatened animals and smaller proportions of protected area estate because of a stronger desire to prioritize domestic socioeconomic and security interests above environmental protection and participation global challenges, such as biodiversity conservation (Hale, 2020; Hodgetts et al., 2019).

## METHODS

### Model development

We used a model selection procedure to assess the importance of national regime ideology as a predictor of threatened species and protected area estate alongside other predictor variables (Appendix S1) generated based on theoretical considerations or known from previous work to influence biodiversity outcomes (Tables 1 & 2). In generating models based on theoretical grounds, we considered 3 theories widely applied to global biodiversity loss investigations: ecological modernization theory (McKinney et al., 2009), treadmill of production theory (Hoffmann, 2004), and world polity theory (Shandra et al., 2010). We also included models informed by previous studies that considered governance (Baynham‐Herd et al., 2018), democracy (Rydén et al., 2020), and economic inequality (Kubiszewski et al., 2023). This approach allowed us to examine whether regime ideology was a significant factor for biodiversity outcomes on its own or whether regime ideology was more influential through the lens of other known correlates of biodiversity loss. For example, if gross domestic product (GDP) predicted threatened species, would the effect be consistent across economies of different ideological philosophies?

**TABLE 1:** Theoretical justification and data used to predict biodiversity outcomes (proportion of animals threatened and proportion of land area designated as protected area estate).

Model^a^	Theoretical link to biodiversity	Variables in model (averaged from 2005 to 2009)	Sources for theoretical link
Ecological modernization theory	Biodiversity outcomes will improve as countries become more technologically advanced and adopt postmaterialistic proenvironmental agendas.	Gross domestic product (GDP) per capita (ln); population urbanization	Hoffmann, 2004; McKinney et al., 2010; Mol, 1996
Treadmill of production theory	Perpetual economic growth demands continually opening new frontiers into ecological destruction and will decrease biodiversity outcomes.	GDP (ln); GDP growth rate	Habibullah et al., 2022; Hoffmann 2004
World polity theory	Integration into the global community will improve biodiversity outcomes as countries adopt a “world environmental agenda” where proenvironmental values and resources are diffused internationally.	Konjunkturforschungsstelle (KOF) globalization index	Baynham‐Herd et al., 2018; Shandra et al., 2009
Democracy	Democracy will improve biodiversity outcomes because of increased scientific transparency and governmental accountability.	Varieties of Democracy (V‐Dem) electoral democracy index	Rydén et al., 2020
Governance	Governance will improve biodiversity outcomes because of better regulatory enforcement, higher trust in government policy, and lower levels of political corruption that could jeopardize environmental protection.	World Bank governance indicators (average of all indicators used)	Amano et al., 2018; Baynham‐Herd et al., 2018; Smith et al., 2003
Inequality^b^	Inequality will decrease biodiversity outcomes because it restricts the collective action required to address national environmental issues.	Gini index	Holland et al., 2009; Mikkelson et al., 2007

^a^ Saturated (all variables) and null (no variables) models included in analyses. Each model was tested with and without regime ideology variables (Table 2) included to examine the relative influence of regime ideology on model performance. For all models (except inequality), the sample size was 165 countries.

^b^ Tested separately due to substantially smaller sample size for Gini index (*n* = 117).

**TABLE 2:** Ideological and spatial variables included in the analysis of biodiversity outcomes (proportion of animals threatened and proportion of land area designated as protected area estate).

Variable	Prediction	Variables to test prediction (averaged from 2005 to 2009^a^)	Sources used for prediction
Conservative Regime Ideology	Conservative regimes will be associated with higher proportions of threatened animals and greater protected area estate.	V‐Dem regime ideology character variable reported as “restorative or conservative”	Apostolopoulou et al., 2021; Penca, 2013
Socialist Regime Ideology	Socialist regimes will be associated with lower proportions of threatened animals and less protected area estate.	V‐Dem regime ideology character variable reported as “socialist or communist”	Baldauf, 2020; Kopnina et al., 2018
Nationalist Regime Ideology	Nationalist regimes will be associated with higher proportions of threatened animals and less protected area estate.	V‐Dem regime ideology character variable reported as “nationalist”	Hale, 2020; Hodgetts et al., 2019
Land Area	Larger land area will be associated with greater numbers of species and more opportunity for protected area establishment.	Land area (km^2^) transformed to natural log	Hoffmann, 2004
Latitude and Longitude^b^	Used to test for effects of spatial autocorrelation.	Longitude and latitude (taken from the geographical centroid of each country)	Fletcher & Fortin, 2018

*Note*: To each of the models in Table 1, we included the ideology character variables (conservative, socialist, and nationalist) to test if model performance was improved relative to the same model without regime ideology. Land area was included in all threatened animal models as a predictor variable and in all protected area models as an offset variable.

^a^ Only applicable to regime ideology variables.

^b^ Spatial autocorrelation analysis performed on saturated models prior to running the set of candidate models.

### Ecological modernization theory

Ecological modernization theory posits that as economies grow and become more advanced, their opportunities and willingness for positive ecological outcomes will also increase (Hoffmann, 2004; Mol, 1996). The ecological modernization model embraces economic growth and development whereby “the only way out of the ecological crisis is going further into modernization” (Mol, 1995, p. 42). For example, more advanced economies (postindustrialized) typically have access to superior biodiversity monitoring tools (e.g., thermal drone imagery) and expertise (e.g., the ability to perform complex veterinary procedures). The theory, which has been subject to a variety of criticisms, predicts advanced economies can reorientate themselves along ecologically sensitive lines, pursuing economic growth and ecological protection simultaneously and ultimately decoupling resource use and economic production (Frame, 2022; McKinney et al., 2010). Ecological modernization can be inferred from measures of affluence, including per capita wealth or the percentage of the population living in urban areas (Hoffmann, 2004; McKinney et al., 2009).

### Treadmill of production theory

The treadmill of production theory is often used to counter ecological modernization. Like ecological modernization, efficiency gains and technological innovations are still expected; however, those efficiency gains and innovations open the door to new investments and production methods, driving further economic expansion and ecological decline (Jorgenson, 2016; Schnaiberg, 1980). The result is that economies can become stuck on a metaphorical “treadmill of production” where the quality of life is no longer improved by economic growth, yet negative ecological impacts of constant economic expansion continue to increase (Curran, 2017; Gould et al., 2004). The treadmill of production threatens biodiversity because production increases require natural capital and sustained activities such as deforestation, mining, and pollution (Gould et al., 2004; Hoffmann, 2004). The treadmill of production can be inferred from national economic metrics, including total economy size or economic growth rate (Habibullah et al., 2022; Hoffmann, 2004).

### World polity theory

World polity theory posits that participation in the international community connects nations through globalization. Globalization helps diffuse values and objectives for global social, economic, and environmental problems and encourages international cooperation (Gareau & Lucier, 2018). World polity explains how international organizations, such as the United Nations (UN) and the International Union for Conservation of Nature (IUCN), help to define and deliver the global biodiversity agenda while accelerating the adoption of conservation as a globally shared value (Baynham‐Herd et al., 2018; Gareau & Lucier, 2018). World polity works to disincentivize biodiversity loss and uses the risk of international reputational damage to drive environmental treaty commitments and greater conservation action (McKinney et al., 2010). World polity has previously been associated with lower levels of threatened species and reduced forest loss (Shandra et al., 2009, 2010, 2011). World polity has been inferred from various measures ranging from the number of international organizations operating in the country to general globalization indices (Baynham‐Herd et al., 2018; Shandra et al., 2009).

### Other important factors

In addition to the 3 theories mentioned above, and given their links to biodiversity outcomes, we included models for governance (Barrett et al., 2006), inequality (Kubiszewski et al., 2023), and democracy (Rydén et al., 2020). Based on previous work, more robust governance was expected to improve biodiversity outcomes because it can be synonymous with better enforced protected areas, lower rates of corruption, which can jeopardize environmental protection, and higher public trust in environmental policy (Amano et al., 2018; Baynham‐Herd et al., 2018; Smith et al., 2003). Democracy was expected to improve biodiversity outcomes because of increased governmental transparency, accountability, and separation of power (Rydén et al., 2020). Finally, economic inequality was expected to impair biodiversity outcomes because countries with high inequality are more likely to have populations unable to participate in the collective action required to conserve nature, given that their basic needs are often unmet (Holland et al., 2009; Mikkelson et al., 2007).

### Data sources

For our outcome variables, we first used the IUCN Red List data set 2022‐02. We took the total number of threatened (critically endangered + endangered + vulnerable) animal species per country (IUCN, 2023). From the same IUCN Red List data set, we also collected data for the total number of animal species per country. Second, we used data from Protected Planet accessed in November 2023 to obtain each country's total terrestrial and inland‐water protected area coverage (measured in km^2^) (UNEP‐WCMC & IUCN, 2023).

We used data from The Varieties of Democracy (V‐Dem) Project to account for regime ideology (Coppedge et al., 2023). We used 3 ideology character variables that classified the national government ideology as nationalist, socialist, and conservative (among others), based on expert opinions, on a scale ranging from 0 (expert consensus on absence of the ideology) to 1 (expert consensus on presence of the ideology) (Tannenberg et al., 2019). We treated the consensus among country experts that a government promotes a specific ideology as a general measure of how strong the ideology is within the national regime. All GDP data were obtained from the Varieties of Democracy database, and we took the natural log of GDP and GDP per capita values (Coppedge et al., 2023; Fariss et al., 2022). Urbanization data were obtained from the World Bank (World Bank, 2023). Globalization was measured via the Konjunkturforschungsstelle (KOF) globalization index provided by the KOF Swiss Economic Institute as an indicator of a nation's participation in the global community (Gygli et al., 2019). As a measure of inequality within a nation, we used the Gini index of income inequality from the World Bank, which ranges from 0 (absolute equality) to 1 (absolute inequality) (World Bank, 2023). To measure governance, we followed previous studies by averaging the 6 governance dimensions (control of corruption, government effectiveness, political stability, rule of law, regulatory quality, and voice and accountability) provided by the World Bank and made available on the Varieties of Democracy database (Baynham‐Herd et al., 2018; Coppedge et al., 2023; World Bank, 2023). Democracy was measured via the varieties of democracy electoral democracy index, which is a generalized indicator of democracy that reflects transparent, inclusive, open, and fair national electoral processes (Coppedge et al., 2023). We also collected data for the total land area of each country provided by the World Bank (World Bank, 2023). Last, to investigate the potential that our outcome measures were spatially autocorrelated, we collected data on the latitude and longitude of each country, taken at the geographical centroid, from *The World Factbook* (CIA, 2021).

### Time lag

**FIGURE 1:**
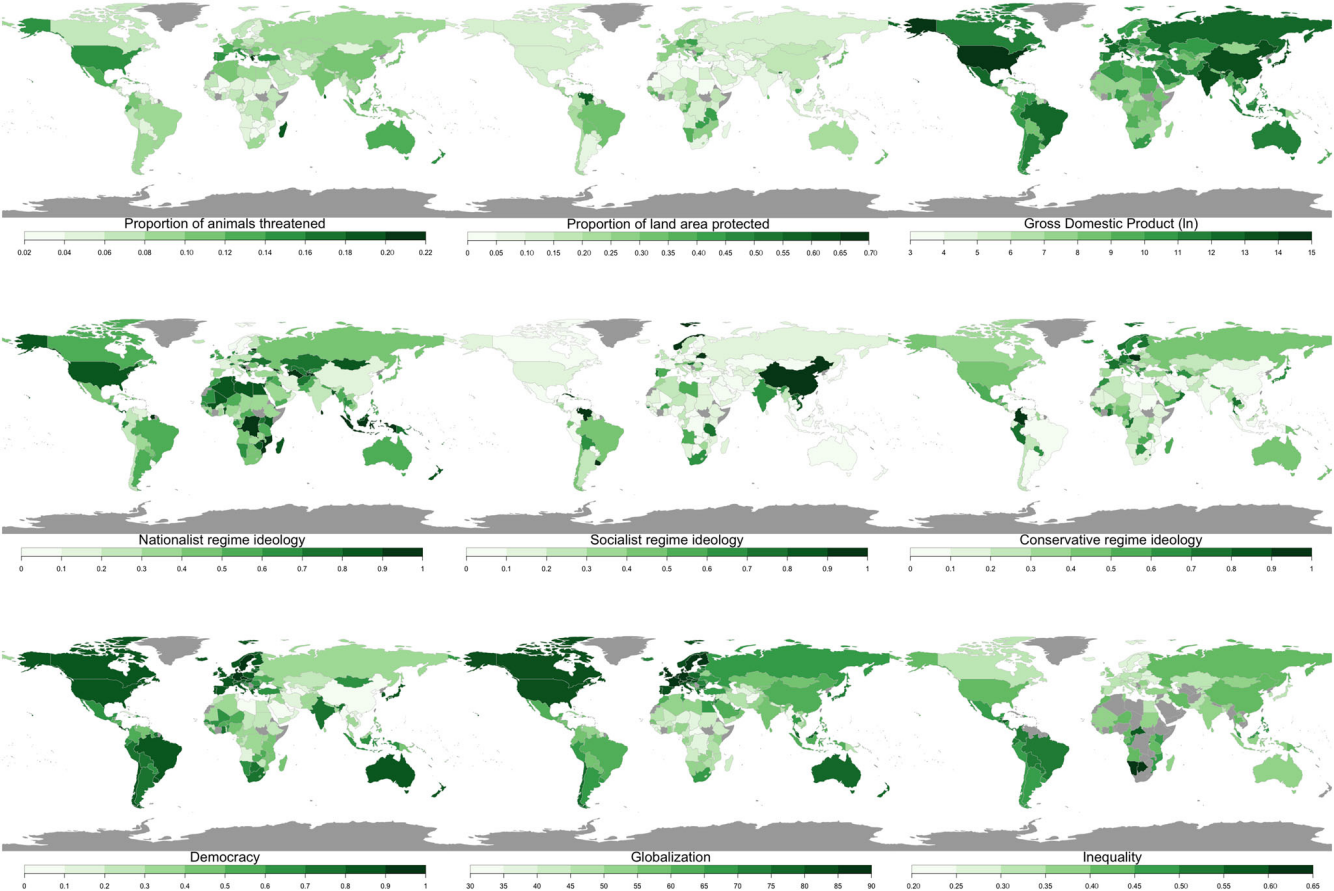
Data used in the negative binomial regression analysis to predict biodiversity outcomes (not all variables shown) (gray, countries with missing data). Proportion of animals threatened and proportion of land area protected data are from 2023. All other variables are averaged over 2005–2009. Data for gross domestic product (GDP) were obtained via the Varieties of Democracy (V‐Dem) Project database and transformed to the natural log. Regime ideology variables are provided by V‐Dem. Metrics: democracy, V‐Dem electoral democracy index; globalization, Konjunkturforschungsstelle Globalization Index provided by the Swiss Economic Institute; inequality, the Gini index of income inequality (0, equality; 1, perfect inequality) provided by the World Bank. The maps prepared with the R package rworldmap (South, 2023).

It is widely acknowledged that it takes approximately 15 years for socioeconomic forces to materialize into measurable changes in biodiversity outcomes (Hoffmann, 2004; Holland et al., 2009; Mikkelson et al., 2007; Shandra et al., 2009). Because we took advantage of the opportunity to include recent IUCN Red List threatened species data and statistics on protected area establishment, we chose 2007 to measure our predictor variables. However, because our data were collected from various international sources that range in availability and reporting frequency, not all variables provided data for the year 2007. To address this problem, we followed previous studies and took the mean score over 5 years for every predictor variable between 2005 and 2009 (Holland et al., 2009; Figure 1). Taking the average score over 5 years allowed us to increase our sample size to 165 countries (excluding the Gini index, where *n* = 117). Additionally, in any given year, many nations will experience ideological shifts resulting from elections and regime changes. Therefore, taking the 5‐year average for regime ideology allowed us to capture ideological trends better.

### Statistical analyses

To determine the relevance of regime ideology for national biodiversity outcomes, we ran a series of generalized linear models with a negative binomial distribution and a log link function for threatened animals and protected area estate separately, using the R package MASS (Ripley, 2023). For the threatened animal models, we used each country's total animals as an offset variable and controlled for total land area by including it as a predictor variable. For the protected area models, we set each country's land area as the offset variable. The use of offsets essentially made both outcome variables a proportion and helped for making an equitable analysis between countries (Hoffmann, 2004). For both outcome variables, we considered 7 model specifications (i.e., ecological modernization, treadmill of production, world polity, governance, democracy, a saturated model, and a null model) with and without the regime ideology variables included. This resulted in a candidate set of 14 models for both dependent variables. We omitted the inequality models from the primary analysis due to the substantially smaller sample size. However, we reran models with the smaller data set separately to examine inequality.

To mitigate the risk of collinearity in the models, we examined variance inflation factors (VIFs) and sequentially removed predictor variables with a VIF >5 (Zuur et al., 2009). We initially ran saturated models with all predictor variables included for both threatened animals and protected areas; however, VIFs for the globalization and GDP per capita were >5 and therefore were omitted from both models. However, globalization and GDP per capita were still included in their respective models where VIF scores remained acceptable (Tables 3 & 4). The fit of the saturated models was assessed with the R package DHARMa (Hartig, 2022), which indicated a good distribution of model residuals for both threatened animals and protected areas. For every model, we calculated McFadden's pseudo *R* ^2^ and Akaike's information criterion corrected for small sample sizes (AICc) to rank the models. The lowest AICc represented the overall best model that maximized model fit while minimizing the number of predictor variables to encourage parsimony (Mazerolle, 2006; Portet, 2020; Symonds & Moussalli, 2010). Additionally, we ran a model selection function from the R package MuMIn (Barton ´, 2023) to calculate the relative weight of evidence in support of each model from the set of 14 candidate models for both threatened animals and protected areas (Tables 3 & 4).

**TABLE 3:** Ranked negative binomial generalized linear model coefficients, significance, and 95% confidence intervals (in parentheses) shown for the proportion of animals listed as threatened by the International Union for Conservation of Nature (IUCN) in 165 countries.

Predictor variable	Treadmill of production + ideology	Treadmill of production	Saturated model + ideology	Saturated model	World polity	World polity + ideology	Ecological modernization + ideology	Ecological modernization	Governance	Governance + ideology	Democracy	Null model	Democracy + ideology	Regime ideology
Land Area (ln)	−0.056^***^	−0.053^***^	−0.064^***^	−0.063^***^	0.013	0.012	0.021	0.021	0.022	0.022	0.015	–	0.016	0.015
	(−0.088 to −0.024)	(−0.085 to −0.022)	(−0.100 to −0.028)	(−0.100 to −0.026)	(−0.013 to 0.039)	(−0.013 to 0.038)	(−0.006 to 0.047)	(−0.006 to 0.048)	(−0.006 to 0.050)	(−0.005 to 0.050)	(−0.013 to 0.042)		(−0.012 to 0.043)	(−0.013 to 0.042)
GDP (ln)	0.120^***^	0.116^***^	0.128^***^	0.128^***^	–	–	–	–	–	–	–	–	–	–
	(0.087 to 0.154)	(0.082 to 0.149)	(0.088 to 0.169)	(0.087 to 0.169)										
GDP per capita (ln)	–	–	–	–	–	–	0.118^**^	0.127^**^	–	–	–	–	–	–
							(0.037 to 0.198)	(0.046 to 0.207)						
GDP growth rate	−0.176	−0.198	−0.254	−0.298	–	–	–	–	–	–	–	–	–	–
	(−0.516 to 0.176)	(−0.545 to 0.162)	(−0.646 to 0.148)	(−0.699 to 0.113)										
Urbanization	–	–	0.042	−0.038	–	–	−0.013	−0.102	–	–	–	–	–	–
			(−0.264 to 0.349)	(−0.346 to 0.271)			(−0.427 to 0.402)	(−0.518 to 0.314)						
Globalization	–	–	–	–	0.009^***^	0.010^***^	–	–	–	–	–	–	–	–
					(0.006 to 0.013)	(0.006 to 0.014)								
Democracy	–	–	0.012	−0.042	–	–	–	–	–	–	0.224	–	0.220	–
			(−0.310 to 0.331)	(−0.371 to 0.285)							(−0.005 to 0.452)		(−0.011 to 0.450)	
Governance	–	–	−0.049	−0.032	–	–	–	–	0.108^**^	0.105^**^	–	–	–	–
			(−0.159 to 0.060)	(−0.142 to 0.079)					(0.039 to 0.178)	(0.034 to 0.177)				
Nationalism	0.371^**^	–	0.376^**^	–	–	0.312^*^	0.346^*^	–	–	0.295	–	–	0.299	0.276
	(0.109 to 0.633)		(0.109 to 0.642)			(0.034 to 0.590)	(0.057 to 0.635)			(0.004 to 0.586)			(0.003 to 0.594)	(−0.023 to 0.575)
Socialism	0.293^*^	–	0.302^*^	–	–	0.284^*^	0.332^*^	–	–	0.292^*^	–	–	0.315^*^	0.320^*^
	(0.033 to 0.554)		(0.039 to 0.566)			(0.008 to 0.562)	(0.048 to 0.617)			(0.003 to 0.583)			(0.023 to 0.609)	(0.023 to 0.619)
Conservatism	0.196	–	0.221	–	–	0.168	0.233	–	–	0.233	–	–	0.300	0.339^*^
	(−0.083 to 0.475)		(−0.062 to 0.504)			(−0.133 to 0.470)	(−0.071 to 0.538)			(−0.082 to 0.548)			(−0.015 to 0.614)	(0.024 to 0.655)
Constant	−3.237	−2.881	−3.248	−2.817	−3.230	−3.534	−3.348	−2.970	−2.760	−3.076	−2.799	−2.503	−3.153	−3.026
Highest VIF	1.713	1.515	3.668	3.568	1.001	1.708	2.878	2.802	1.056	1.707	1.009	–	1.710	1.702
McFadden *R* ^2^	0.166	0.162	0.166	0.162	0.151	0.154	0.152	0.149	0.145	0.147	0.142	0.140	0.145	0.143
AICc	1764.0	1765.6	1769.7	1770.9	1785.5	1786.2	1792.3	1792.4	1799.2	1800.7	1804.7	1805.0	1805.5	1806.7
Akaike weight	0.648	0.292	0.039	0.021	0.000	0.000	0.000	0.000	0.000	0.000	0.000	0.000	0.000	0.000

*Note*: Models ranked by Akaike information criterion corrected for small sample size (AICc). Significance: *p < 0.05; **p < 0.01; ***p < 0.001. All models include total animals per country listed by IUCN as an offset variable.

Abbreviations: GDP, gross domestic product; ln, natural log transformed; VIF, variance inflation factor.

**TABLE 4:** Ranked negative binomial generalized linear model coefficients, significance, and 95% confidence intervals (in parentheses) shown for the proportion of land area listed as protected area by Protected Planet in 165 countries.

Predictor variable	Democracy	Democracy + ideology	Governance	Saturated model	Saturated model + ideology	Governance + ideology	World polity	World polity + ideology	Treadmill of production	Treadmill of production + ideology	Regime ideology	Null model	Ecological modernization	Ecological modernization + ideology
GDP (ln)	–	–	–	−0.033	−0.039	–	–	–	−0.008	−0.020	–	–	–	–
				(−0.097 to 0.032)	(−0.102 to 0.025)				(−0.067 to 0.051)	(−0.079 to 0.039)				
GDP per capita (ln)	–	–	–	–	–	–	–	–	–	–	–	–	0.110	0.095
													(−0.051 to 0.270)	(−0.068 to 0.258)
GDP growth rate	–	–	–	−0.173	−0.185	–	–	–	−0.869^*^	−0.841^*^	–	–	–	–
				(−0.948 to 0.656)	(−0.953 to 0.635)				(−1.519 to −0.155)	(−1.502 to −0.116)				
Urbanization	–	–	–	−0.110	−0.180	–	–	–	–	–	–	–	−0.182	−0.216
				(−0.739 to 0.524)	(−0.810 to 0.453)								(−1.056 to 0.692)	(−1.093 to 0.660)
Globalization	–	–	–	–	–	–	0.008^*^	0.007	–	–	–	–	–	–
							(0.001 to 0.016)	(−0.001 to 0.014)						
Democracy	0.880^***^	0.839^***^	–	0.800^*^	0.777^*^	–	–	–	–	–	–	–	–	–
	(0.437 to 1.321)	(0.393 to 1.283)		(0.101 to 1.481)	(0.096 to 1.443)									
Governance	–	–	0.191^**^	0.049	0.057	0.178^**^	–	–	–	–	–	–	–	–
			(0.062 to 0.322)	(−0.157 to 0.255)	(−0.154 to 0.267)	(0.046 to 0.313)								
Nationalism	–	0.000	–	–	−0.060	0.018	–	−0.012	–	−0.031	0.024	–	–	0.018
		(−0.627 to 0.626)			(−0.689 to 0.566)	(−0.628 to 0.662)		(−0.670 to 0.647)		(−0.687 to 0.627)	(−0.640 to 0.688)			(−0.653 to 0.685)
Socialism	–	0.427	–	–	0.451	0.469	–	0.395	–	0.416	0.422	–	–	0.432
		(−0.174 to 1.032)			(−0.147 to 1.050)	(−0.153 to 1.095)		(−0.235 to 1.031)		(−0.212 to 1.049)	(−0.215 to 1.064)			(−0.202 to 1.072)
Conservatism	–	0.548	–	–	0.530	0.538	–	0.550	–	0.631^*^	0.696^*^	–	–	0.609
		(−0.114 to 1.209)			(−0.126 to 1.186)	(−0.144 to 1.221)		(−0.153 to 1.253)		(−0.051 to 1.313)	(0.007 to 1.386)			(−0.091 to 1.309)
Constant	−2.172	−2.456	−1.671	−1.736	−1.898	−1.993	−2.182	−2.382	−1.423	−1.643	−2.053	−1.677	−1.811	−2.102
Highest VIF	–	1.712	–	2.977	3.108	1.708	–	1.707	1.007	1.717	1.706	–	2.719	2.809
McFadden *R* ^2^	0.089	0.091	0.088	0.090	0.091	0.089	0.087	0.088	0.087	0.089	0.087	0.086	0.086	0.088
AICc	3667.6	3668.8	3673.8	3674.4	3675.0	3675.0	3677.0	3678.6	3678.7	3678.8	3679.7	3680.0	3681.2	3682.2
Akaike weight	0.586	0.323	0.027	0.020	0.015	0.015	0.005	0.002	0.002	0.002	0.001	0.001	0.001	0.000

*Note*: Models ranked by Akaike information criterion corrected for small sample size (AICc). Significance: *p < 0.05; **p < 0.01; ***p < 0.001. All models include total country land area (km^2^) as an offset variable.

Abbreviations: GDP, gross domestic product; ln, natural log transformed; VIF, variance inflation factor.

Last, cross‐national ecological and socioeconomic studies are highly susceptible to the effects of spatial autocorrelation, which can inflate the risk of type 1 error in model interpretation (Claessens et al., 2023; Fletcher & Fortin, 2018). We calculated a distance matrix of neighboring countries up to the first 13,285 km based on great circle distances and the latitude and longitude at the geographical centroid of each country with the R package *spdep* (Bivand, 2023). This distance was based on the rule of thumb whereby spatial correlation analysis should be limited to two thirds of the greatest distance between points of interest, which was 19,927 km for our study (Fletcher & Fortin, 2018). Based on the neighbor matrix, we simulated global Moran's *I* for the residuals of both saturated models via 9999 Monte Carlo permutations to provide an overall spatial correlation statistic. Out of an abundance of caution, we also plotted spline correlograms with the R package *ncf* (Bjornstad, 2022) to visually determine whether model residuals at specific distances apart were spatially correlated. Following visual inspection of the correlograms, we resimulated global Moran's *I* statistics at distances of interest by manually changing the neighbor distance threshold.

## RESULTS

For both saturated models, the overall spatial autocorrelation statistic, global Moran's *I*, was small and statistically insignificant (threatened animals: Moran's *I* = −0.0025, *p* = 0.0897; protected areas: Moran's *I* = −0.0053, *p* = 0.3044). The supplementary analysis with spline correlograms indicated no more than a weak spatial autocorrelation (|Moran's *I*| < 0.15) for the threatened animal model residuals up to the first 1800 km and therefore did not exceed the threshold for statistical intervention (Fletcher & Fortin, 2018; O'Sullivan & Unwin, 2010). The spline correlogram for the protected area model residuals did not suggest the presence of spatial autocorrelation at any distance. Thus, we did not consider spatial autocorrelation further.

For threatened animals, the inclusion of regime ideology improved model performance among the best candidate models. The model selection procedure yielded the strongest support for treadmill of production theory, indicating that greater economic activity drives higher proportions of threatened animals as indicated by the positive and significant GDP model coefficient (Table 3). The treadmill of production model including regime ideology variables (Akaike weight = 0.648, McFadden *R* ^2^ = 0.166) had over double the weight of evidence as the best candidate model compared to the same model without ideology (Akaike weight = 0.292, *R* ^2^ = 0.162). In this model, nationalist ideology (β = 0.371, *p* < 0.01) and socialist ideology (β = 0.293, *p* < 0.05) had positive model coefficients and were significant. The 2 models considering treadmill of production theory had a combined Akaike weight of 0.94, suggesting there was a 94% probability that, of the models tested, one of these 2 models was the best. The saturated models with ideology (Akaike weight = 0.039) and without ideology (Akaike weight = 0.021) comprised the remaining 6% of possible support. Similarly, the inclusion of ideology approximately doubled the probability of the saturated model being the best candidate model. No other models tested registered meaningful evidence of support (Table 3).

**FIGURE 2:**
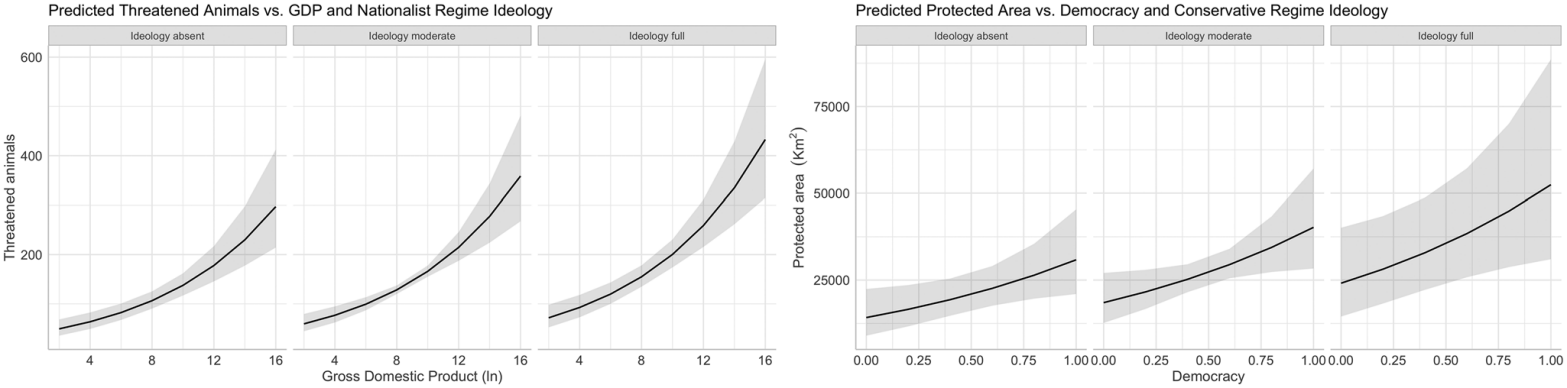
Predicted values (marginal effects) of threatened animals (left) and protected area estate (km^2^) (right) from the saturated negative binomial generalized linear models (shading, 95% confidence interval; *x*‐axis, most significant predictor variable [i.e., lowest *p* value] calculated for representative levels of the most relevant regime ideology type based on expert consensus; 0, absence of the ideology; 0.5, moderate presence of ideology; 1, full presence of the ideology) (Tannenberg et al., 2019).

Nationalist and socialist regime ideologies were statistically significant predictors of threatened animals across most candidate models, indicating they are somewhat more relevant to threatened animals compared with conservative regime ideology. Across all threatened animal models, regime ideology model coefficients were positive, suggesting that stronger presence of any ideology within nation states may contribute to higher proportions of threatened animals. Regime ideology did not make valuable contributions to models concerning ecological modernization, world polity, democracy, or governance (Table 3). Regime ideology by itself also performed relatively poorly, suggesting that the effects of ideology are more relevant in association with other factors, such as GDP (Figure 2).

For the protected area models, regime ideology was less relevant. Of the 14 candidate models, the democracy model was the best (Akaike weight = 0.58;6, McFadden *R* ^2^ = 0.089) (Table 3). The next best performing model was democracy plus regime ideology (Akaike weight = 0.323, *R* ^2^ = 0.091); positive model coefficients suggested that stronger democracy (β = 0.839, *p* < 0.001) and conservative regime ideology (β = 0.548, *p* < 0.1) were associated with higher proportions of protected area estate. Like the threatened animal candidate models, a single perspective (i.e., democracy, and democracy + ideology) yielded approximately 90% probability of containing the best model, and the effects of regime ideology appeared most relevant in association with other factors, such as democracy (Figure 2). However, unlike the threatened animal models, the inclusion of regime ideology generally halved, rather than doubled, the Akaike weight of the better models. Across all protected area candidate models, conservative regime ideology was the only ideology predictor variable to achieve statistical significance at α < 0.05, although this occurred in only 2 models (Table 4). There was also evidence to suggest governance was an important factor to consider in the relative establishment of protected areas (Table 4).

Finally, to investigate the effects of regime ideology and inequality, we reran models with a smaller sample size (*n* = 117) to include the Gini index. However, the Gini index was not a statistically significant predictor of either threatened animals or protected area estate. Furthermore, the inclusion of regime ideology did not result in a lower AICc for either outcome variable, suggesting that considering ideology alongside inequality did not meaningfully improve the model.

## DISCUSSION

To avert the biodiversity crisis, conservation must succeed across all political persuasions, and here we provided the first evidence that national regime ideology may contribute to biodiversity outcomes. By demonstrating a link between regime ideology and biodiversity, conservationists might tailor advocacy and initiatives to consider a government's ideological values in addition to other important factors. We found that GDP and land area were important factors for explaining threatened animals. Positive GDP coefficients suggested that larger economies were associated with an increased proportion of threatened animals. This effect is widely documented and may be because higher GDP can be a broader proxy for natural resource use, land‐use change, infrastructure, and environmental accidents (Czech et al., 2012). For the models with the highest weight of evidence, land area had a negative coefficient, suggesting larger land area was associated with lower proportions of threatened animals. This effect might be explained by the species–area relationship because nations with larger land areas typically contain a greater biodiversity, which can result in more resilient ecosystems (Hoffmann, 2004; Weeks et al., 2022). Overall, the analysis provided the greatest support for the treadmill of production theory, suggesting that larger economies are closely associated with ecological decline.

We found that the weight of evidence in support of the treadmill of production model approximately doubled once regime ideology was considered (Table 3). As hypothesized, nationalist governments were associated with increased proportions of threatened animal species once GDP size and growth were considered. Nationalism often emphasizes domestic individualism and autonomy, which can be particularly problematic for global environmental issues that require diplomatic and economic cooperation between nations through sharing responsibility, knowledge, and resources (Hale, 2020; MacDonald, 2003). Nationalist regimes may also be less likely to participate in conservation initiatives that work across the borders of neighboring countries, which could present barriers to transboundary species conservation that will likely only worsen with the effects of climate change (Hale, 2020; Titley et al., 2021). Future global conservation efforts should be mindful of the effect of nationalist regime ideology when designating conservation resources or should employ targeted strategies to integrate better with nationalist ideological perspectives. For example, conservation organizations might emphasize biodiversity conservation as a matter of national interest rather than global interest. This could include the pride in conserving iconic native species and natural heritage, portraying conservation as a matter of national security through environmental and social prosperity or focusing on the domestic economic benefits of conserving biodiversity (Hodgetts et al., 2019).

Contrary to our expectations, socialist regime ideology was also positively associated with an increased proportion of threatened animals (Table 3). However, the effects of socialist regimes on biodiversity outcomes are likely to occur for different reasons compared with nationalist ideology. Although a good portion of literature theorizes socialist ideology is more environmentally orientated, there is still little historical evidence that socialist regimes have been better for nature than other ideologies (Dominick, 1998; Kopnina et al., 2018; Tickle, 2010). Our results paint a similar picture. For example, socialism has still relied on industrial development and the use of natural resources to raise living standards, which can risk prioritizing rapid development over sustainable development and increase the frequency of human–wildlife conflict through intervening factors, such as deforestation (Horning, 2008; Kluvánková‐Oravská et al., 2009). In contrast to nationalist ideology, global conservation interventions may best integrate with socialist ideology by advocating the human benefits of biodiversity conservation. This might include an emphasis on cultural value, human health, intergenerational equality, and global citizenship (Baldauf, 2020; Domínguez & Luoma, 2020; Hayward et al., 2022; Kopnina et al., 2018).

A different set of predictor variables was associated with protected area estate versus threatened animals. This was likely because the threatened animal model measured the physical threat to biodiversity and the protected area model measured national commitment to mitigating biodiversity loss. Although we expected to find the strongest support for world polity theory and thus see globalization explain relative protected area establishment (which is regularly reported by countries in international forums), it was democracy that emerged as the most important factor (Table 4). Democracy, like globalization, is associated with higher governmental accountability and transparency. According to Baynham‐Herd et al. (2018), world polity might still occur indirectly through building greater capacity for conservation through democratic institutions. However, democracy has previously shown mixed results in predicting biodiversity conservation outcomes, including protected area establishment (Rydén et al., 2020). Our results provided evidence that democracy is an important factor for explaining national protected area estate. Although democracy was important for explaining protected areas, it was not as relevant for explaining the number of threatened animal species in a country (Table 3). These findings might imply a misalignment between the political conditions required for government conservation action and the conditions ideal to physically reduce species loss. For example, the lack of long‐term planning and funding for many protected areas has compromised their integrity and performance and has led to the rise of paper parks (Agrawal & Ostrom, 2006; Dasgupta, 2021).

Conservative regime ideology was the only ideology variable to achieve statistical significance in the protected area candidate models, although this occurred only twice (Table 4). This finding partially aligned with our expectations that conservative ideology would be associated with more traditional conservation interventions. Conservative ideology has the strongest connection to capitalism and neoliberal conservation paradigms, which can benefit conservation by placing barriers between society and wilderness, creating ideal conditions for activities such as ecotourism or biodiversity offsetting (Apostolopoulou et al., 2021; Penca, 2013). However, like democracy, although conservative regime ideology was linked to greater protected area establishment, there was no evidence to suggest it predicted threatened animals (Table 3).

Although our findings provide the first evidence that regime ideology may be relevant to future biodiversity conservation planning, as with any novel perspective, we only scratched the surface of this relationship in our investigation. As such, we draw attention to some limitations of our study and suggest directions that future research could take to help shed further light on the important association between national regime ideology and biodiversity. First, although we took a cross‐sectional approach, there is a need for longitudinal research to quantify how regime ideology has influenced biodiversity outcomes throughout different geopolitical eras. Second, our analyses did not include interaction effects or many of the potential intervening factors, such as energy use, deforestation, or a breakdown of economic activity into goods and services, that are often operationalized by regime ideology and may have more direct relationships with biodiversity outcomes. Third, although we demonstrated that democracy and conservative regime ideology were relevant factors for explaining protected area establishment, protected areas are only one part of the solution to the biodiversity crisis (Santangeli et al., 2023). Future studies could examine the relationship between regime ideology and national biodiversity funding, or the number of conservation policies implemented. Finally, future research could consider the structural inequalities between developed and developing nations. In cross‐national biodiversity research, this concept is often operationalized via world systems theory or ecological unequal exchange theory (Frey et al., 2019). These theories posit developing countries, often rich in biodiversity, act as both a global source of natural resources and as a sink for associated waste and ecological damage due to structural inequalities of global trade and geopolitical systems (Hoffmann, 2004; Jorgenson, 2016). Regime ideology plays an important role in constructing and maintaining these global systems (Büscher & Fletcher, 2014; Hodgetts et al., 2019).

Nevertheless, with this study, we are the first to quantify the links between national regime ideology and biodiversity outcomes at the cross‐national scale. Specifically, we found that GDP, land area, nationalist regime ideology, and socialist regime ideology were important predictors of the relative number of threatened animals in a country. These results have implications for global conservation efforts and open a new frontier to designing more successful conservation interventions. By demonstrating the link between regime ideology and biodiversity, conservation organizations and policy makers might develop policies and programs to resonate better with a government's ideological values. For example, existing norms of promoting biodiversity conservation as an act of global citizenship to a nationalist government is unlikely to be an effective strategy. Instead, conservation interventions might be promoted as critical to national security or domestic economic prosperity (Hodgetts et al., 2019). Alternatively, for socialist governments, conserving biodiversity for the benefit of people is more likely to be an effective advocacy strategy (Kopnina et al., 2018). We also presented strong evidence that democracy and, to a lesser extent, conservative regime ideology are relevant factors for explaining relative protected area establishment. However, the same associations between democracy and conservative regime ideology and the number of threatened animals were not evident. Ultimately, there is no one‐size‐fits‐all approach to designing effective global conservation interventions, and increasing efforts to tailor the right policy to the right audience is essential.

## Supporting information

Supporting information.
